# BRCA1 Mutation Leads to Deregulated Ubc9 Levels which Triggers Proliferation and Migration of Patient-Derived High Grade Serous Ovarian Cancer and Triple Negative Breast Cancer Cells

**Published:** 2016-06-23

**Authors:** J Xu, A Footman, Y Qin, K Aysola, S Black, V Reddy, K Singh, W Grizzle, S You, D Moellering, ES Reddy, Y Fu, VN Rao

**Affiliations:** 1 Cancer Biology Program, Department of OB/GYN, Morehouse School of Medicine, Georgia Cancer Center for Excellence, Grady Health System, Atlanta, USA; 2 Department of Pathology, University of Alabama at Birmingham, Birmingham, USA; 3 VA Medical Center, Emory University School of Medicine, Georgia; 4 Department of Nutrition, University of Alabama at Birmingham, Birmingham, USA

**Keywords:** BRCA1, BRCA1a, Ubc9, Serous Epithelial Ovarian Cancers, Triple Negative Breast Cancers, Protein-Protein Interaction, Migration, Proliferation

## Abstract

Women who carry a germline mutation in BRCA1 gene typically develop triple negative breast cancers (TNBC) and high grade serous ovarian cancers (HGSOC). Previously, we reported that wild type BRCA1 proteins, unlike the disease-associated mutant BRCA1 proteins to bind the sole sumo E2-conjugating enzyme Ubc9. In this study, we have used clinically relevant cell lines with known BRCA1 mutations and report the *in-vivo* association of BRCA1 and Ubc9 in normal mammary epithelial cells but not in BRCA1 mutant HGSOC and TNBC cells by immunofluorescence analysis. BRCA1-mutant HGSOC/TNBC cells and ovarian tumor tissues showed increased expression of Ubc9 compared to BRCA1 reconstituted HGSOC, normal mammary epithelial cells and matched normal ovarian tissues. Knockdown of Ubc9 expression resulted in decreased proliferation and migration of BRCA1 mutant TNBC and HGSOC cells. This is the first study demonstrating the functional link between BRCA1 mutation, high Ubc9 expression and increased migration of HGSOC and TNBC cells. High Ubc9 expression due to BRCA1 mutation may trigger an early growth and transformation advantage to normal breast and ovarian epithelial cells resulting in aggressive cancers. Future work will focus on studying whether Ubc9 expression could show a positive correlation with BRCA1 linked HGSOC and basal like TNBC phenotype.

## Introduction

Women who have mutations in the BRCA1 gene have an increased lifetime risk of developing hereditary breast and ovarian cancers. BRCA1 mutations are responsible for up to 10% of the epithelial ovarian cancers (EOC) and 15% of breast cancers [1, 2]. Epithelial ovarian cancers are typically of the serous subtype and can be subdivided into two categories: Type I and Type II tumors. Type I is considered a low-grade tumor, whereas type II is considered a high-grade tumor; type II is the most malignant form of ovarian cancer accounting for up to 70% of all ovarian cancer diagnoses [3]. The most common and aggressive histotype of epithelial ovarian cancer (EOC), high grade serous carcinoma (HGSOC) is associated with germ line BRCA1 mutations with a lifetime risk of 40–60% [4]. Over 75% of breast tumors found in women with a BRCA1 mutation have a so-called triple negative phenotype (TNBC), meaning that these tumors do not express estrogen receptor, progesterone receptor and human epidermal growth factor receptor type 2 (HER2) [5]. A high percentage of BRCA1-associated hereditary and sporadic breast cancers are triple negative and are more likely to occur among pre-menopausal women of African American descent [6]. For women of African American descent there is a low survival rate for individuals that have advanced EOC or TNBC due to lack of treatment for advanced epithelial ovarian cancer and no targeted treatment for TNBC. Therefore, there is a critical need for better targeted therapies for these high grade TNBC and ovarian cancers [7–9].

We have identified and cloned two major splice variants of BRCA1, namely BRCA1a/p110 and BRCA1b/p100 [10, 11], both are expressed at lower levels in ovarian and breast tumors as opposed to normal cells [12–15]. Although, the mechanism of tumor suppression remains unknown, we found BRCA1a protein to induce apoptosis and inhibit *in vivo* tumor growth of CAL51 TNBC and hormone-independent ES-2 ovarian cancer cells [16, 17]. BRCA1 promoter hyper methylation has been identified as an important mechanism for BRCA1 inactivation in sporadic breast cancer and appears to correlate with reduced BRCA1 mRNA and protein. Recent integrated analyses of messenger RNA expression, microRNA expression, DNA methylation and DNA copy number aberrations have shown that more than 30% of high-grade serous ovarian carcinomas and basal-like breast cancers had a dysfunctional BRCA pathway as a consequence of germline or somatic BRCA1/2 mutations or BRCA1 promoter hyper methylation [5]. BRCA1 and its splice variants are nuclear proteins that contain several functional domains, an N-terminal RING finger domain that interacts with several proteins and two-BRCA1 C-terminal domains involved in transcriptional activation. BRCA1, BRCA1a and BRCA1b proteins are nuclear-cytoplasmic shuttling proteins that are also localized in the mitochondria [10, 15, 18, 19]. The action of nuclear localization signals (NLS) and nuclear export signals (NES) located in the RING domain that mediates nuclear transport via association with BARD1 are also responsible for the regulation of BRCA1 nuclear transport [20]. The BRCA1 delta isoform, which lacks NLS, also enters the nucleus via the RING-domain mediated BARD1 import pathway [21]. The RING domain of BRCA1, in complex with BARD1, mediates an E3 Ubiquitin ligase activity on ER-α *in-vitro* [21, 22]. Using an Ubiquitin ligase-deficient BRCA I26A mutant, recent findings suggest that the Ubiquitin ligase activity is expendable for both, genomic stability and homology-directed repair of double-strand DNA breaks, however the Ubiquitin ligase activity is essential for repression of ER-α activity [23, 24].

Many proteins are known to undergo post-translational modifications which play a major role in regulating gene expression [25]. SUMO (Small Ubiquitin-like modifier) modification of proteins is a dynamic and reversible process that affects several functions like stability, localization, protein-protein interactions and transcriptional regulation [26–28]. The SUMO modification pathway was shown to be involved in BRCA1 response to DNA damage and transcriptional repression [29, 30]. We have shown the amino-terminal domain of BRCA1, BRCA1a and BRCA1b proteins to bind to SUMO-E2-conjugating enzyme Ubc9 and regulate ER-α activity by promoting its degradation *in vivo* [31]. This work suggested that there is a cross talk between the SUMO and Ubiquitin pathways, similar to the Ubiquitin ligase RNF4, by highlighting a new biochemical function of BRCA1 as a putative SUMO-1 and Ubc9-dependent E3 Ubiquitin ligase for ER-α SUMO conjugates [32, 33]. Ubc9 binding site mutations, as well as cancer-predisposing mutation in the BRCA1 RING domain (C61G), disrupted the ability to modulate Ubc9-mediated estrogen-induced ER-α transcriptional activity in breast cancer cells [31] but did not disrupt SUMO-1 binding [29] nor auto ubiquitination activity of BRCA1 [31]. Both BRCA1/BRCA1a K109R and disease associated C61G mutants, which are localized mainly in the cytoplasm, fail to suppress the growth of TNBC and ovarian cancer cells [34]. Ubc9 has been shown to play an important role in both tumor progression and resistance to chemotherapy [35–38]. In fact, Ubc9 was found to act as both a positive and negative regulator of proliferation and transformation of HMGA1 proteins [39]. Here, we have further investigated these findings in physiologically relevant BRCA1 germ line mutant TNBC and HGSOC cell lines obtained from patients. Using these cells we have studied the *in vivo* association of BRCA1 with Ubc9, expression of Ubc9 in these BRCA1 mutant TNBC and HGSOC cell lines and tumor tissues. We have also studied the effect of knock-down of Ubc9 on proliferation and migration of these cells. Our data suggests SUMOylation pathway to be a potentially important candidate for targeted therapy for BRCA1 associated TNBC and HGSOC.

## Materials and Methods

### Cell Culture

MCF10A, HCC1937, UWB1.289 and UWB1.289 BRCA1 cells were obtained from American Type Culture Collection (Rockville, MD, USA) and cultivated as described previously [34, 40, 41] HCC1937 cells were grown in RPMI 1640 medium with 20% FBS and 1% PS.

### Western blot analysis

MCF10A, HCC1937, UWB1.289 and UWB1.289 BRCA1 cells were seeded into 10 cm Petri-dishes with a density of 2 × 10^6^. After 48 hours the cell pellets were lysed in SUMO lysis buffer (62.5 mM Tris-HCl, pH 6.8, 2% SDS) and the proteins were separated on 4–20% gradient SDS-PAGE and transferred to nitrocellulose membrane. The primary antibodies for BRCA1 (Calbiochem, AB-1, mAb, 1:100), Ubc9 (Abcam, ab21193, pAb, 1:1000), Ubc9 N-15 and β-Actin (Santa Cruz C4, 1/1000) were used to probe the proteins of interest. The protein bands were visualized by Image Reader LAS-3000 (FUJIFILM) using HRP labeled secondary antibody to mouse or goat and developing solution (GE Healthcare). The signal of Ubc9 protein band was quantified using software MultiGauge. The values are standardized with the internal control β-Actin, UWB1.289 is defined as 1. Bars represent the mean ± SE of at least two experiments. For western blot analysis using patient tissue samples, Ubc9 protein levels were screened in seven patient sets of papillary adenocarcinomas/normal adjacent tissue lysates using INSTA-blot ovary tissue OncoPair from INGENEX. For the blot the tissue specimens were homogenized in modified RIPA buffer to obtain the soluble proteins and centrifuged to clarify. Sample buffer was added to the soluble fraction and approximately 14 μg/lane of protein was run and then transferred to PVDF membrane. The blot was probed with the Ubc9 primary antibody (Santa Cruz N15, 1/100) and HRP labeled secondary antibody to goat and developing solution (GE Healthcare). The bands were visualized by Image Reader LAS-3000 (FUJIFILM) as before.

### Antibodies and reagent

The antibodies used in this study were MS110 ascites (Ab1, EMD Chemicals), Ubc9 N-15, β-Actin antibodies (Santa Cruz Biotechnology) and Ubc9 ab21193 (Abcam).

### Immunofluorescence microscopy

MCF10A, HCC1937 and UWB1.289 and UWB1.289 BRCA1 cells were cultured in six-well plates onto glass coverslips overnight. The cells were washed and fixed in icy methanol for 5 minute, and blocked using 10% BSA for 60 min, followed by primary polyclonal Rabbit anti-Ubc9 antibody 1:150, Monoclonal Mouse anti-BRCA1 antibody 1:100 diluted in 1.5 % BSA made in PBS at 25°C 1hr and Alexa488 goat anti-Rabbit/Alexa568 goat anti-mouse (Molecular Probes) diluted in 1.5% BSA/PBS for 50 min and stained (Hoechst 33258, Pentahydrate, Life technologies). The cover slips were mounted with Vectashield mounting medium for fluorescence (H-1000 from Vector). The stained cells were examined by LSM 700 Confocal Microscope, equipped with 63× oil Ph immersion objectives. Composite filter cubes were used for the 488–405 as described previously [41].

### RT- PCR analysis

UWB1.289 and UWB1.289 BRCA1 cells were seeded into 6-well plates with the same density. Forty-eighty hours later, the total mRNA was extracted with RNeasy Mini Kit (Qiagen) and the first-strand cDNA was synthesized by superscript II Reverse Transcriptase (Invitrogen) and the target cDNA was amplified by PCR with specific primers for either BRCA1 or Ubc9 and visualized by Image Reader LAS-3000 (FUJIFILM). GAPDH was used as the internal control. The signal of Ubc9 RT-PCR was quantified using software MultiGauge. The values are standardized with the internal control GAPDH, UWB1.289 is defined as 1. Bars represent the mean ± SE of at least two experiments.

### siRNA transfection and cell growth analysis

The UWB1.289 cell line was maintained in 50% RPMI and 50% MEGM, supplemented with 3% fetal bovine serum. Before transfection, UWB1.289 cells were digested with 0.25% of Trypsin-EDTA solution and seeded into 6-well plate with a density of 1.5×10^5^/well. Twenty-four hours later, dilute Ubc9 siRNA and control siRNA (Qiagen) in the culture medium without serum (final concentration is 50 nM), HiPerFect Transfection Reagent (Qiagen) was added to the diluted siRNA and mixed by vortexing. Incubated the mixture for 5–10 min at room temperature (15–25°C) to allow the formation of transfection complexes. The complexes were added drop-wise onto the cells and gently swirled to ensure uniform distribution of the transfection complexes. The cells were incubated for 36 hours and images were taken using a fluorescent microscope (Olympus).

### Cell proliferation assay using ECIS

The cell growth assays were done using the ECIS (Applied Biophysics) technology as described by the manufacturer. The UWB1.289 cell line was maintained in 50% RPMI and 50% MEGM, supplemented with 3% fetal bovine serum. The Electric Cell-substrate Impedance Sensing (ECIS) instrument and culture ware 8W10E+ array were purchased from Applied Biophysics (Troy, NY). The cells were grown to reach 80% confluence and the culture medium was removed, briefly rinsed the cell layer with Ca^++^/Mg^++^ free Dulbecco’s phosphate-buffered saline (D-PBS), the cells were digested with 0.25% of Trypsin-EDTA solution and observed under an inverted microscope until cell layer was dispersed (usually within 5 to 15 minutes). The trypsin digestion of the cells was terminated with the complete growth medium aspirated by gentle pipetting and counted. The total cell number of 8×10^4^ of UWB1.289 cells were seeded into the 8W10E+ array and transfected with Ubc9 siRNA or control siRNA (100 nM) (Qiagen) and pre-warmed in the CO_2_ incubator until the temperature of the cell suspension reached 37°C. The array was then connected to the ECIS instrument electrode. The instrument was set to record the cell growth behavior with time course as described by the manufacturer.

### Migration assay

To perform migration assays 2 × 10^5^ ES-2, HCC1937 and CAL51 cells were plated into 6-well cell culture plate. Cells were transfected with 100 nM of Ubc9 siRNA or control siRNA using Qiagen kit as described by the manufacturer. After 24 hours a 1mm wide scratch was made across the cell monolayer using a sterile 200 μl pipette tip. Cells were allowed to grow in normal medium for 24 hours to 48 hours. Plates were photographed at 0 h immediately after scratch and 24 hours or 48 hours following scratching as needed. All experiments were repeated at least twice.

## Results

### Loss of association of BRCA1 and Ubc9 in BRCA1 mutant HGSOC and TNBC cells

**Figure 1:**
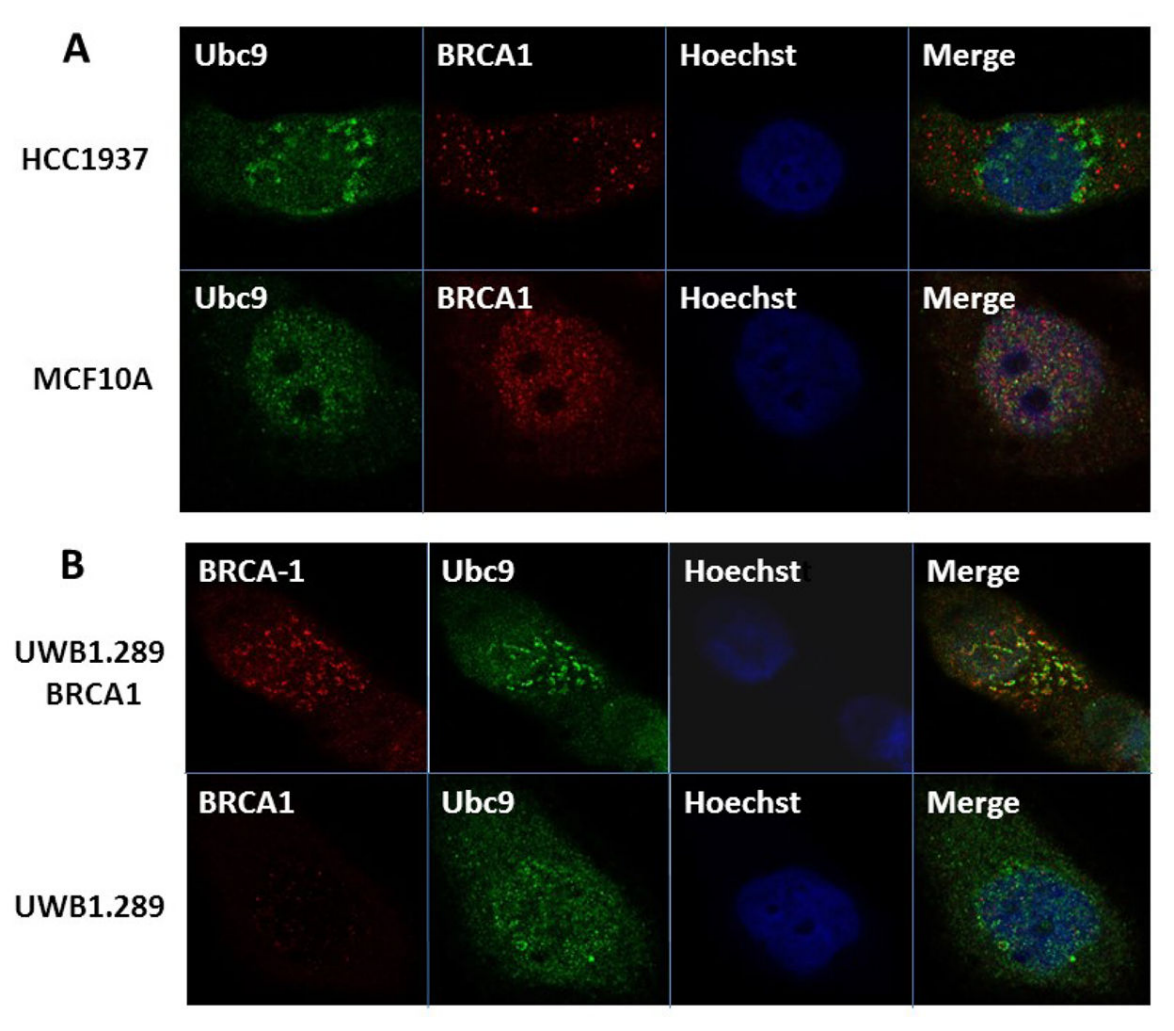
Co-localization of endogenous Ubc9 with endogenous BRCA1 in normal mammary epithelial cells MCF10A and UWB1.289+BRCA1 cells but not in BRCA1 mutant SEOC cells UWB1.289 and BRCA1 mutant TNBC cells HCC1937 ( A and B) by immunofluorescence analysis using anti-UBC9 and anti-BRCA1 Antibodies. MCF10A, HCC1937, UWB1.289, UWB1.289+BRCA1 cells were cultured in six-well plates onto glass coverslips overnight. They were washed and fixed in methanol and blocked using 10% BSA, followed by primary polyclonal Rabbit anti-Ubc9 antibody 1:150 /Monoclonal Mouse anti-BRCA1 antibody 1:100 diluted in 1.5% BSA/PBS at 25°C 1hr and Alexa488 goat anti-Rabbit/Alexa568 goat anti-mouse (Molecular Probes) diluted in 1.5% BSA/PBS and stained with Hoechst33258. The cover slips were mounted with mounting medium. The stained cells were examined by LSM 700 Confocal Microscope, equipped with 63× oil Ph immersion objectives. Composite filter cubes were used for the 488–405.

Wild Type BRCA1 but not the disease associated mutants have been shown to bind *in vitro* to SUMO E2 conjugating enzyme Ubc9 and this is responsible for both ER-alpha activation and tumor suppression of breast and ovarian cancer cells [31, 34]. To examine whether this occurs *in vivo*, we have studied the association of BRCA1 and Ubc9 in normal mouse mammary epithelial cells MCF10A and a basal-like TNBC cell line HCC1937 obtained from a patient with germ line BRCA1 mutation using immunofluorescence analysis. Our results show *in vivo* association of BRCA1 and Ubc9 in MCF10A but not in HCC1937 cells (Figure 1A). Similarly we have studied the *in vivo* association of BRCA1 and Ubc9 in a HGSOC cell line UWB1.289 obtained from a patient with BRCA1 mutation and BRCA1 reconstituted UWB1.289 cells by immunofluorescence analysis using BRCA1 and Ubc9 antibodies. Our results show localization of BRCA1 and Ubc9 in UWB1.289 BRCA1 cells but not in the UWB1.289 cells (Figure 1B). These results support our previous hypothesis that lack of association of BRCA1 with Ubc9 could be responsible for the development of TNBC and HGSOC.

### Ubc9 is expressed at elevated levels in BRCA1 mutant HGSOC, TNBC cells and ovarian tumor tissues

**Figure 2:**
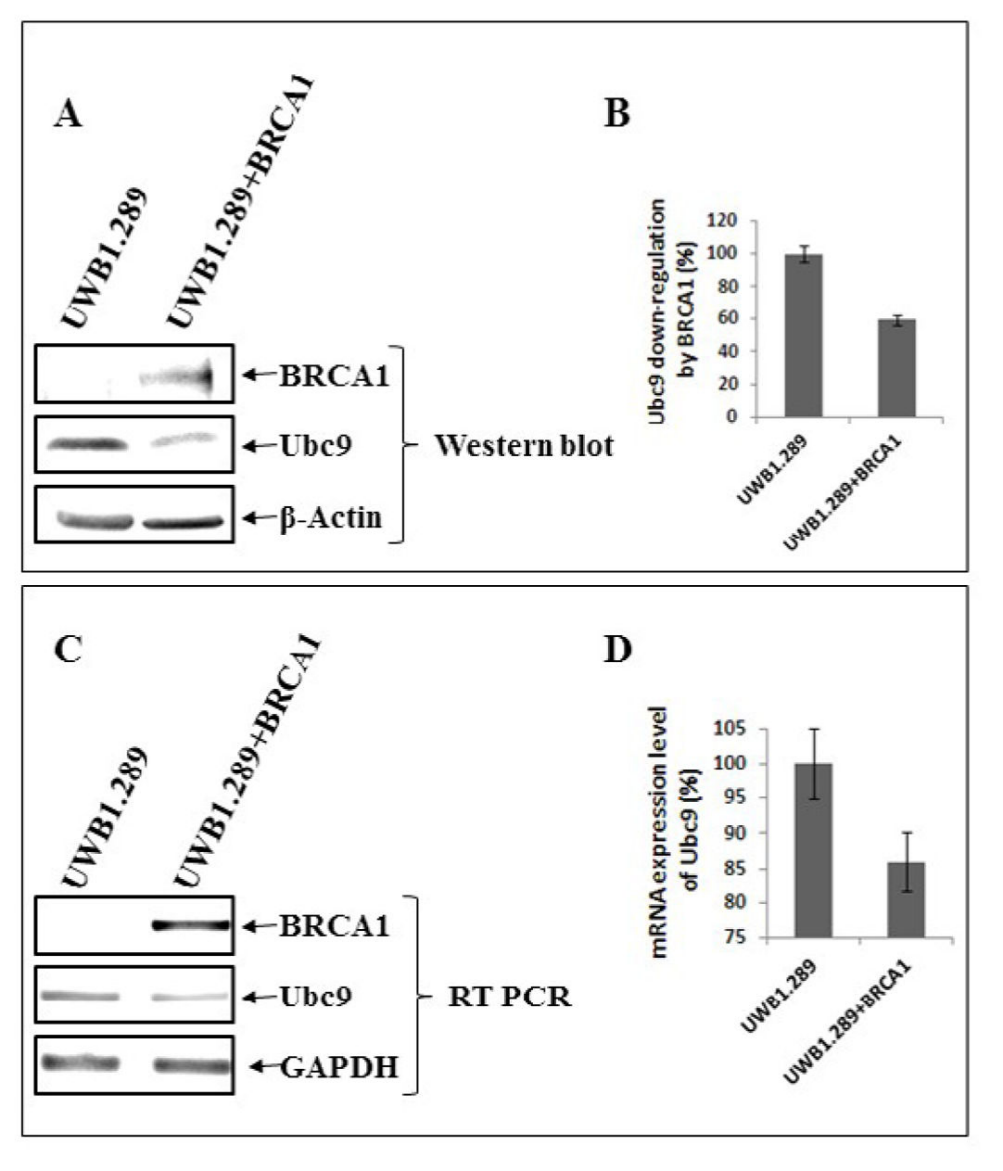
Ubc9 is expressed at elevated levels in BRCA1 mutant SEOC cells UWB1.289 compared to BRCA1 reconstituted UWB1.289 cells. A: Western blot analysis of BRCA1 and Ubc9 in UWB1.289 and UWB1.289 BRCA1 cell lines. The cells were seeded into 10 cm Petri-dishes with a density of 2×10^6^. After 48 hours the cell pellets were lysed in SUMO lysis buffer (62.5 mM Tris-HCl, pH 6.8, 2% SDS) and the proteins were separated on 4–20% gradient SDS-PAGE gel and transferred to the nitrocellulose membrane. The primary antibodies for BRCA1 (Calbiochem, AB-1, mAb, 1:100), Ubc9 (Abcam, ab21193, pAb, 1:1000) and β-Actin (Santa Cruz C4, 1/1000) were used to probe the different proteins of interest. The protein bands were visualized by Image Reader LAS-3000 (FUJIFILM) using HRP labeled secondary antibody to mouse or goat and developing solution (GE Healthcare). B: The stoichiometry of Ubc9 protein levels shown in A. The signal of Ubc9 protein band was quantified using software MultiGauge. The values are standardized with the internal control β-Actin, UWB1.289 is defined as 1. Bars represent the mean ± SE of at least two experiments. C: RT-PCR analysis of mRNA expression of BRCA1, Ubc9 and control GAPDH. The UWB1.289 and UWB1.289 BRCA1 cell lines were seeded into the 6-well plate with the density of 2×10^6^. Forty-eighty hours later, the total mRNA was extracted with RNeasy Mini Kit (Qiagen) and the first-strand cDNA was synthesized by SuperScript II Reverse Transcriptase (Invitrogen) and the target cDNA was amplified by PCR with specific primers of either BRCA1 or Ubc9 and visualized by Image Reader LAS-3000 (FUJIFILM). GAPDH was used as the internal control. D: The stoichiometry of Ubc9 mRNA levels is shown in C. The signal of Ubc9 RT-PCR was quantified using software MultiGauge. The values are standardized with the internal control GAPDH, UWB1.289 is defined as 1. Bars represent the mean ± SE of at least two experiments.

**Figure 3:**
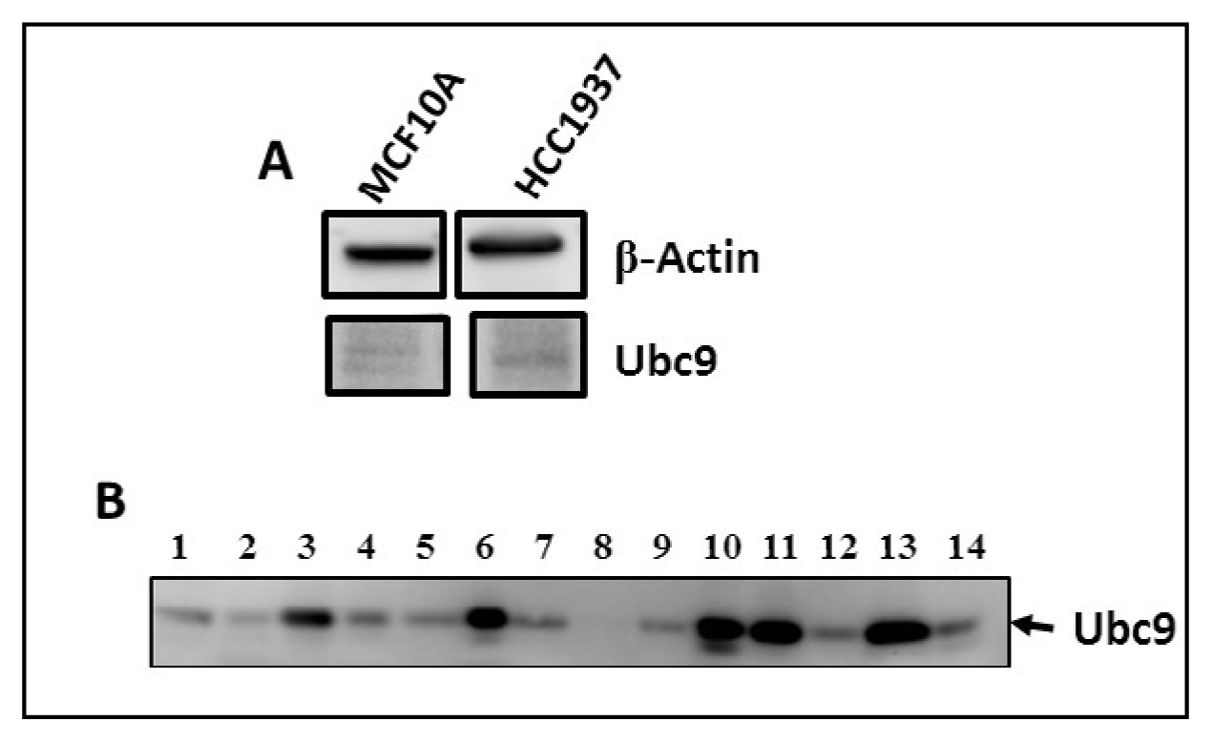
A: Ubc9 is expressed at higher levels in BRCA1 mutant TNBC cells HCC1937 compared to normal mammary epithelial cells MCF10A. Western blot analysis of MCF10 A and HCC1937 cells using Ubc9 and actin antibodies. B: Ubc9 is over expressed in ovarian tumors as compared to matched normal ovarian tissues. Ubc9 protein levels were screened in seven patient sets of papillary adenocarcinomas (lanes 1, 3, 5, 7, 9, 11, 13) and adjacent normal tissue (lanes 2, 4, 6, 8, 10, 12, 14) lysates using INSTA-blot Ovary. Tissue Onco Pair from IMGENEX. The tissue specimens were homogenized in modified RIPA buffer to obtain the soluble proteins and centrifuged to clarify. Sample buffer was added to the soluble fraction and approximately 14 μg/lane of protein was run and then transferred to PVDF membrane. Soluble fraction extraction buffer: PBS at pH 7.4, 1 μg/ml Aprotinin, 1mM NaF modified RIPA buffer: 1mM EDTA, 1 μg/ml pepstatin-A, 0.1% SDS, 0.25% Na deoxychalate, 1 μg/ml Leupeptin, 1mM PMSF, 1 mM Na3VO4. Ubc9 was probed with the Ubc9 primary antibody (Santa Cruz N15, 1/100) and visualized by Image Reader LAS-3000 (FUJIFILM) using HRP labeled secondary antibody to goat and developing solution (GE Healthcare).

To examine whether lack of binding of mutant BRCA1 to Ubc9 in HCC1937 TNBC cells and UWB1.289 HGSOC cells results in upregulation of Ubc9, we investigated Ubc9 expression in these cell lines. For this we subjected UWB1.289 and UWB1.289 BRCA1 cells to western blot analysis using BRCA1 and Ubc9 antibodies. We also isolated RNA from these cells and performed BRCA1 and Ubc9 cDNA amplification by PCR using their respective primers. Our results showed inverse correlation between BRCA1 expression and Ubc9 levels both at the protein as well as RNA level (Figure 2 A, B, C and D). Similarly we studied the expression of Ubc9 in both MCF10A and HCC1937 cells by western blot analysis using Ubc9 antibodies. We find increased Ubc9 expression in HCC1937 cells compared to MCF10A cells (Figure 3 A). We have previously found high Ubc9 expression in breast tumors compared to normal matched tissues [34]. We studied the expression of Ubc9 in seven patient sets of papillary adenocarcinomas and adjacent normal tissues by western blot analysis using Ubc9 antibody. Our results show enhanced expression of Ubc9 in ovarian tumors compared to matched normal tissues (Figure 3 B). This agrees with our results obtained using breast tumor tissue samples [34] and previous studies that documented higher levels of Ubc9 expression in several cancers compared with their normal tissue counterparts [35–38].

### Knockdown of Ubc9 inhibits proliferation and migration of HGSOC and TNBC

**Figure 4:**
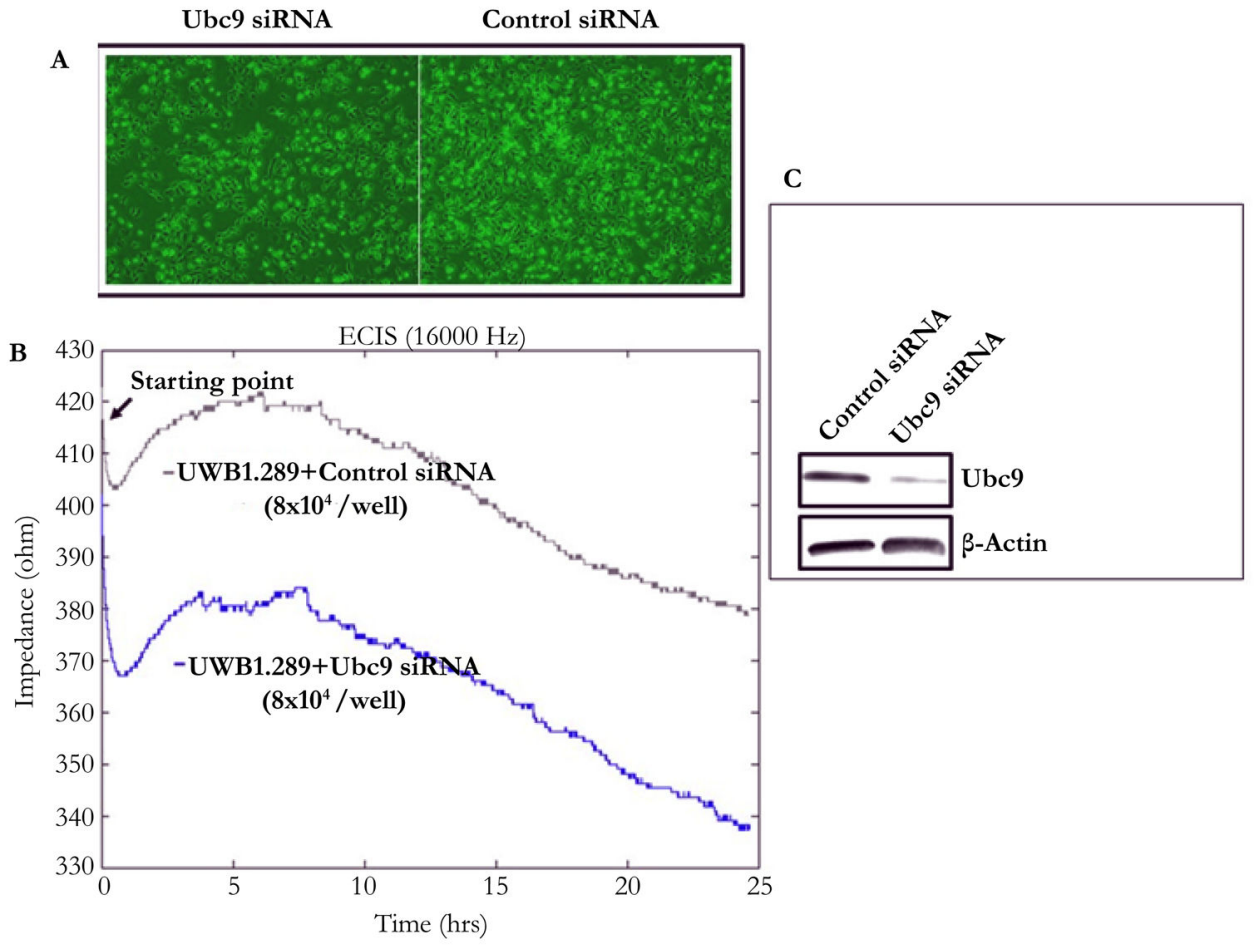
Ubc9 knockdown suppresses the cell growth of ovarian cancer cell line UWB1.289 studied using phase contrast microscope (A) and with (B) Electric Cell-substrate Impedance Sensing (ECIS). The same starting cell density of UWB1.289 cells (shown in graphs) was seeded into the ECIS cultureware 8W10E+ array (Applied BioPhysics) and transfected with Ubc9 or control siRNA (100 nM) (Qiagen). The temperature of the cell suspension was prewarmed to the incubator temperature before the array was connected into the instrument electrode. After Setting up the software and the time course measurement for cell growth was conducted as described by the manufacturer. (C) Western blot analysis showing Ubc9 knockdown in ovarian cancer cell UWB1.289. Ubc9 or control siRNA (Qiagen) were transfected into UWB1.289 cells (1×10^6^) using HiPerFect Transfection Reagent (Qiagen) after cells were seeded into the 10cm of Petri dishes 24 hours. Forty-eight hours later, the cells were collected and lysed in RIPA buffer. The cell lysates were probed with primary antibodies either Ubc9 (Santa Cruz N15, 1/100) or β Actin (Santa Cruz C4, 1/2000). The proteins were visualized using HRP labeled secondary antibody to goat (Ubc9) or mouse (β Actin) and developing solution (GE Healthcare) through Image Reader LAS-3000 (FUJIFILM).

Ubc9 has been shown to be upregulated in multiple cancers and to promote invasion and metastasis in estrogen receptor positive breast cancer cells [35–39]. As mentioned earlier, we observed overexpression of Ubc9 in BRCA1 mutant TNBC cells and HGSOC cells. To study whether downregulation of Ubc9 can inhibit the growth and proliferation of BRCA1 mutant HGSOC cells, we suppressed Ubc9 expression in UWB1.289 cells using siRNA specific to Ubc9 and measured the growth after 36 hours using phase contrast microscope (Olympus). As shown in Figure 4A, Ubc9 knockdown inhibited the growth of UWB1.289 cells unlike the control siRNA transfected cells. We confirmed these results using ECIS (electric cell substrate impedance sensing) assay. ECIS measurements can be used to monitor cell proliferation, and as the cell number increases the amount of electrode area covered with the spread cells grows causing the electrode impedance to rise. These impedance changes can be related to the relative cell proliferation rates. As shown in Figure 4 B Ubc9 knockdown resulted in inhibition of growth when compared to control siRNA transfected UWB1.289 cells, further confirming our previous observations. The expression of was efficiently inhibited by Ubc9 siRNA in UWB1.289 cells as detected by western blot analysis using Ubc9 antibody (Figure 4 C). These results suggest Ubc9 to promote cell proliferation of BRCA1 mutant HGSOC cells.

**Figure 5:**
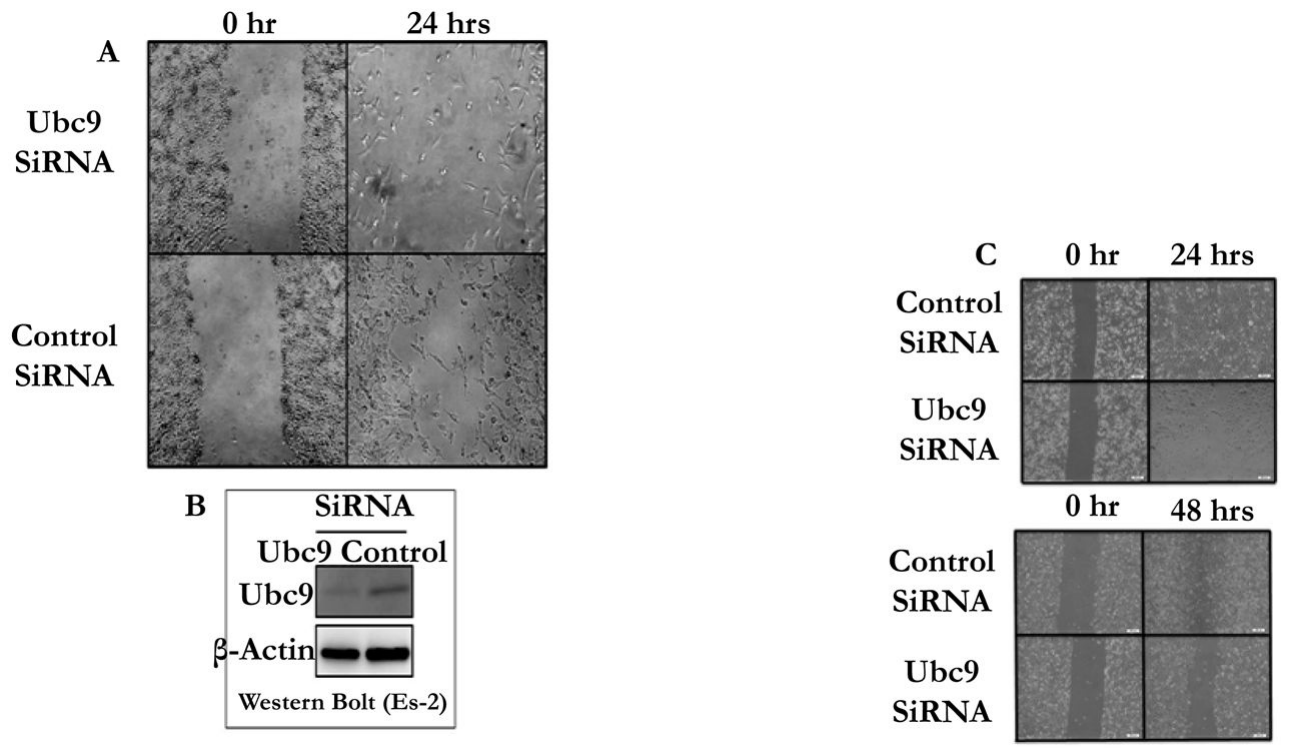
Ubc9 knockdown suppresses the migration of Ovarian and TNBC cells (A, B). ES-2 ovarian cancer cells and (C, D) HCC1937 and CAL51 TNBC cells were seeded into 6-well cell culture plates. Following the transfection with Ubc9 siRNA or control siRNA for 24hrs 1mm wide scratch was made across the cell layer using a sterile pipette tip. Cells were allowed to grow in normal medium for 24 to 48 hrs. Plates were photographed at 0 hr and 24 or 48 hrs after scratching. (B). Western blot analysis of Ubc9 knockdown in ovarian cancer cell ES-2. Ubc9 or control siRNA (Qiagen) were transfected into ES-2 cells (1×10^6^) using HiPerFect Transfection Reagent (Qiagen) after cells were seeded into the diameter 10cm of Petri dishes shortly. After 48 hours of transfection, the cells were collected and lysed in RIPA buffer. The cell lysates were probed with primary antibodies either Ubc9 (Santa Cruz N15, 1/100) or β Actin (Santa Cruz C4, 1/2000). The proteins were visualized using HRP labeled secondary antibody to goat (Ubc9) or mouse (β Actin) and developing solution (GE Health-care) through Image Reader LAS-3000 (FUJIFILM).

To investigate the role of Ubc9 in ovarian and TNBC cell migration we inhibited the endogenous Ubc9 expression using Ubc9 siRNA. As shown in Figure 5 Ubc9 siRNA significantly inhibited the migration of ES-2 ovarian cancer cells (Figure 5A), HCC1937 BRCA1 mutant TNBC cells (Figure 5 C) and CAL-51 a non-BRCA1 mutant TNBC cells (Figure 5 D). Ubc9 expression was shown to be inhibited by Ubc9 siRNA in ES-2 ovarian cancer cells as demonstrated by western blot analysis (Figure 5 B) using Ubc9 antibody. These results strongly emphasize a role for Ubc9 in the migration of ovarian and TNBC cells.

## Discussion

**Figure 6:**
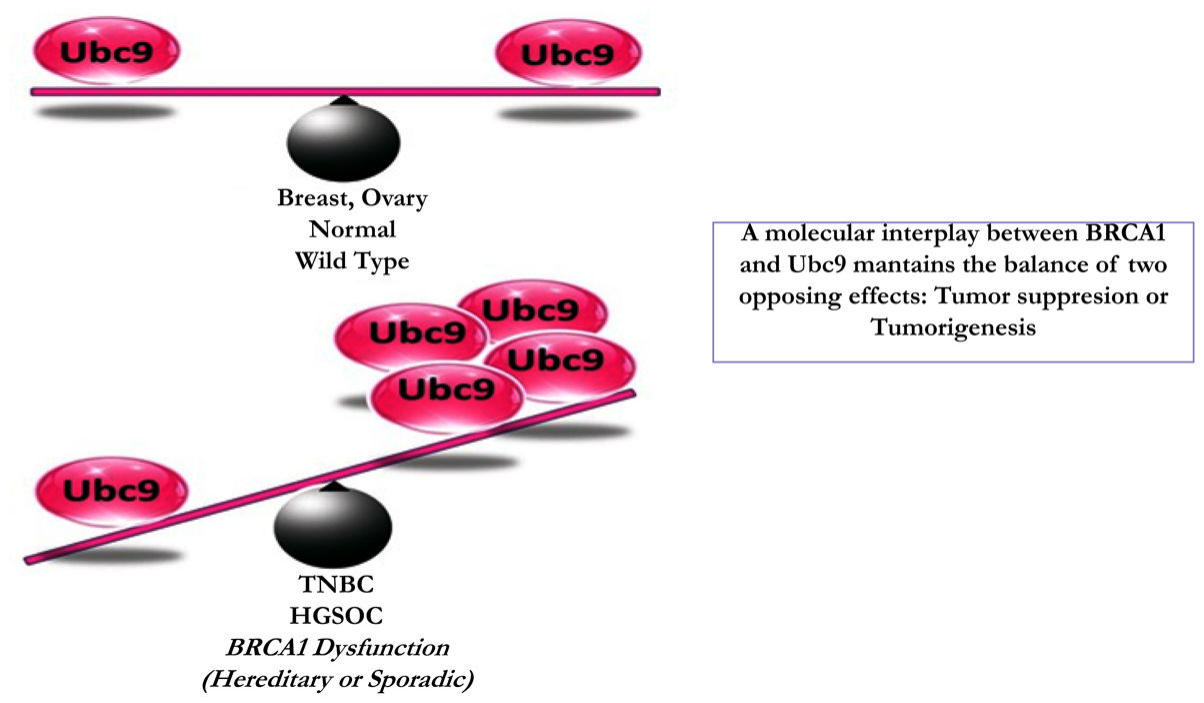
Hypothetical model showing how imbalance in Ubc9 due to BRCA1 mutation or loss of BRCA1 function can trigger the development of TNBC and HGSOC.

SUMOylation is a dynamic protein modification that regulates numerous biological activities of proteins [42, 43]. Increasing studies suggest the SUMO pathway dysregulation in several cancers [44]. Recently, there are several reports connecting protein SUMOylation and cancer. Elevated levels of Ubc9 have been found in several cancers and are associated with poor clinical outcome [45–49]. Our group has previously reported that Wild type BRCA1a/1b proteins unlike the pathogenic mutants to bind Ubc9, a sole SUMO conjugase and perform its function as a tumor suppressor in TNBC and ovarian cancer cells [34, 41]. Although Ubc9 is ubiquitously expressed in normal cells, it was shown to be over-expressed in ovarian, lung, head and neck, melanoma and breast cancers [35–38, 44]. In fact, Ubc9 expression was found to correlate with poor clinical outcome in Nigerian women with breast cancer [50]. Here we have used two physiological relevant patient derived cell lines obtained from BRCA1 mutant TNBC and HGSOC and have shown loss of association of BRCA1 proteins with Ubc9 in these cells unlike normal mammary epithelial cells. Furthermore, we have demonstrated that this results in elevated levels of expression of Ubc9 both at the RNA as well as protein levels in these BRCA1 Ubc9 mutant UWB1.289 ovarian cancer and HCC1937 TNBC cells. We have also found that knockdown of endogenous Ubc9 using siRNA resulted in suppression of cell proliferation and migration of Brca1 mutant TNBC and ovarian cancer cells. In summary, we have shown Ubc9 to be required for cancer cell growth and migration. These results suggest a molecular interplay between BRCA1 and Ubc9 which maintains the balance of two opposing effects: tumor suppression or tumorigenesis. Imbalance in Ubc9 levels due to BRCA1 mutation can tilt this balance resulting in cancer (Figure 6). BRCA1 is a master regulator which by turning off or on Ubc9 binding regulates the normal growth of a cell. These results are consistent with the model that a direct association of BRCA1 with Ubc9 is critical for growth/tumor suppression by BRCA1 proteins and lack of binding results in deregulated Ubc9 levels causing cancer. Ubc9 has been shown to bind to HMGA1 proteins and integrate both positive and negative signals for proliferation and transformation [39]. Recently, Ubc9 was shown to promote cell invasion and metastasis of breast cancer cells [51] implicating a role in tumorigenesis. Several inhibitors of Ubc9 have been reported although none are currently in clinical trials [52–55]. Future work will address whether TNBC and HGSOC with BRCA1 mutations can be selectively eliminated using drugs that target Ubc9.
